# Recent Advance and Application of Wearable Inertial Sensors in Motion Analysis

**DOI:** 10.3390/s25030818

**Published:** 2025-01-29

**Authors:** Laura Gastaldi, Elisa Digo

**Affiliations:** Department of Mechanical and Aerospace Engineering, Politecnico di Torino, 10129 Turin, Italy

The rapid spread of Inertial Measurement Units (IMUs) has revolutionized human motion analysis, providing significant advantages over traditional systems. Low cost, ease of use, unlimited acquisition range, and unobtrusiveness represent only some of the many positive aspects of IMUs. In particular, their ability to conduct motion analysis in ecological environments makes IMUs extremely versatile in a wide range of contexts: (i) the evaluation of patients’ clinical outcomes with particular emphasis on tele-rehabilitation and remote monitoring; (ii) the enhancement of athletic performance and the prevention of injury in sport; and (iii) the improvement of safety and performance in human–robot interaction, guaranteeing more efficient and reliable collaborative systems. Moreover, new applications and challenges are possible thanks to the massive progress achieved by IMUs in terms of miniaturization, performance, and integration into other systems. For example, the combination of IMUs with artificial intelligence techniques, such as machine learning and deep learning, enables precise and accurate recognition of human activities in several scenarios. The Special Issue *Recent Advance and Application of Wearable Inertial Sensors in Motion Analysis* collects eighteen high-quality publications focused on new advances and applications of wearable inertial sensors in different fields. Among these papers, fourteen original scientific articles have been published to fill literature gaps in a meaningful way and, hence, expand the knowledge in the field. In addition, the Special Issue’s collection also includes two systematic reviews summarizing the current expertise and two communications providing new perspectives.

Due to its central role in daily life, gait represents one of the main human movements analyzed through the use of IMUs for different purposes. For example, trunk acceleration patterns recorded through IMUs provide valuable insights for characterizing gait complexity in both healthy and pathological subjects, regardless of age and gait speed (contribution 1). Indeed, these patterns represent a robust tool for comprehensive gait analysis because they reflect the clinical status and kinematic gait abnormalities. However, the exploitation of IMUs during gait can be extended beyond strictly clinical settings to daily healthcare and activity monitoring applications (contribution 2). Wearable inertial sensors are highly effective in pointing out the relationship between variability in gait performance and physical functioning and physical activity (contribution 3). This approach offers a valuable strategy for assessing the risk of falls in older adults with or without pathologies (contribution 4). Moreover, the development of IMU-based algorithms for the real-time detection of gait events facilitates an accurate assessment of the spatio-temporal parameters of gait, not only for healthy subjects (contribution 5). but also for patients wearing an electromechanical orthosis (contribution 6). Considering other useful roles of IMUs, gait analysis can be enhanced by providing an accurate reconstruction of the foot trajectory, including vertical displacement, and identifying terrain types during locomotion (contribution 7). As an alternative, wearable inertial sensors are suitable for understanding the impact of epidural analgesia on maternal mobility, walking patterns, and associated obstetrical outcomes in the first stage of labor (contribution 8). If combined with other technologies, IMUs can contribute to the assessment of the cardiovascular stress risk during prolonged gait (contribution 9). In accordance with the guidelines of this Special Issue, two other publications demonstrate how gait can benefit not only from the use of IMUs, but also from the integration of artificial intelligence. In detail, knee and ankle kinematics can be accurately predicted for each phase of gait through the combined use of IMUs and a deep learning architecture (contribution 10). Another example is represented by the combination of smartphone inertial sensors and a random forest-based algorithm, which can effectively identify transitions between different subtasks of a specific gait test in a lower-limb amputee population (contribution 11).

Beyond gait, other movements and related techniques are also involved in the advancements in wearable technologies. For instance, the angular kinematics of dynamic actions involving both the upper and lower bodies can be effectively assessed by IMUs (contribution 12 and 13). Additionally, fitness tracker watches represent a proper solution to analyze the return to running of non-professional runners after experiencing asymptomatic or mild COVID-19 (contribution 14). Smartwatch IMUs combined with functional calibration algorithms offer an optimal assessment of balance-related metrics (contribution 15). When human motion analysis needs to be performed in environments with ferromagnetic interference, the automatic detection of magnetic disturbances from magnetic IMUs is a crucial goal that can be achieved with deep learning (contribution 16). Finally, artificial intelligence also plays a central role in human activity recognition for gestures regarding both the upper (contribution 17) and the lower (contribution 18) limbs.

In summary, the publications in this Special Issue mark a significant step forward in the field of human motion analysis using wearable inertial sensors. We would like to express our sincere gratitude to all the authors and reviewers who have contributed to this Special Issue, as well as to the staff of the journal *Sensors* for the valuable support.

